# Rickettsial seropositivity in the indigenous community and animal farm workers, and vector surveillance in Peninsular Malaysia

**DOI:** 10.1038/emi.2017.4

**Published:** 2017-04-12

**Authors:** Kai Ling Kho, Fui Xian Koh, Lailatul Insyirah Mohd Hasan, Li Ping Wong, Masoumeh Ghane Kisomi, Awang Bulgiba, Quaza Nizamuddin Hassan Nizam, Sun Tee Tay

**Affiliations:** 1 Department of Medical Microbiology, Faculty of Medicine, University of Malaya, 50603 Kuala Lumpur, Malaysia; 2 Julius Centre University of Malaya, Department of Social and Preventive Medicine, Faculty of Medicine, University of Malaya, 50603 Kuala Lumpur, Malaysia; 3 Department of Veterinary Services, Ministry of Agriculture and Agro-Based Industry Malaysia, Federal Government Administrative Centre, 62630 Putrajaya, Malaysia

**Keywords:** Peninsular Malaysia, Rickettsia, seropositivity, vector surveillance

## Abstract

Rickettsioses are emerging zoonotic diseases that are often neglected in many countries in Southeast Asia. Rickettsial agents are transmitted to humans through exposure to infected arthropods. Limited data are available on the exposure of indigenous community and animal farm workers to the aetiological agents and arthropod vectors of rickettsioses in Peninsular Malaysia. Serological analysis of *Rickettsia conorii* and *Rickettsia felis* was performed for 102 individuals from the indigenous community at six rural villages and 87 workers from eight animal farms in Peninsular Malaysia in a cross-sectional study. The indigenous community had significantly higher seropositivity rates for *R. conorii* (*P*<0.001) and *R. felis* (*P*<0.001), as compared to blood donors from urban (*n*=61). Similarly, higher seropositivity rates for *R. conorii* (*P*=0.046) and *R. felis* (*P*<0.001) were noted for animal farm workers, as compared to urban blood donors. On the basis of the sequence analysis of *gltA*, *ompA* and *ompB*, various spotted fever group rickettsiae closely related to *R. raoultii*, *R. heilongjiangensis*, *R. felis*-like organisms, *R. tamurae*, *Rickettsia* sp. TCM1, *R. felis*, *Rickettsia* sp. LON13 and *R. hulinensis* were identified from tick/flea samples in animal farms, indigenous villages and urban areas. This study describes rickettsial seropositivity of the Malaysian indigenous community and animal farm workers, and provides molecular evidence regarding the presence of rickettsial agents in ticks/fleas infesting domestic animals in Peninsular Malaysia.

## INTRODUCTION

Rickettsioses are emerging infectious diseases that are often neglected in the tropical region. The causative agents, spotted fever group (SFG) and typhus group (TG) rickettsiae, are obligate intracellular bacteria that are transmitted to humans through arthropod vectors, mainly ticks, fleas, mites and so on.^1^ *Rickettsia conorii*, *R. sibirica*, *R. japonica*, *R. honei*, *R. heilongjiangensis*, *R. tamurae* and *R. raoultii* are tick-borne SFG rickettsiae that have been reported in Asia,^2^ whereas flea-borne rickettsioses are usually caused by *R. typhi* and *R. felis*.^3^

Infections caused by SFG rickettsiae and *R. felis* may present as acute febrile illnesses burdening many populations in Southeast Asia. In a Malaysian serological survey conducted 15 years ago, SFG rickettsioses (previously known as tick typhus) have been reported as the most frequent infection among febrile hospitalized patients in rural areas of Peninsular Malaysia. The antibody prevalence of SFG rickettsiae (*R. honei*, TT118 strain) varied widely from 1.7% in urban blood donors to 42.5% in rural febrile patients.^4, 5^ In addition, a high rickettsial seropositivity rate (~50.0%) was also reported among rubber estate workers,^6^ suggesting the endemicity of the disease in this region. It is postulated that people may acquire rickettsioses through exposure to infected ticks and fleas in the living and working environment.

People who live at the fringe of the forest or rural areas, including the indigenous community and animal farm workers, are regarded as populations who are at high risk of acquiring rickettsioses.^7^ The indigenous community in Malaysia (also referred as Orang Asli or ‘original people') constitutes a minority group (0.6%) of the total population in Malaysia.^8^ They stay in huts or settlements that are surrounded by primary or secondary forest, and engage in activities involving agriculture, hunting and collection of forest products. Their nomadic lifestyle and close contact with peri-domestic animals have increased the risk of contracting scrub typhus,^9^ and potential tick- and flea-borne diseases. However, animal farm workers are at risk of multiple tick-borne diseases due to frequent exposure to ticks either from the animals or vegetation.^10, 11^ Few studies have investigated the extent of exposure of these populations to rickettsioses in Southeast Asia. In addition, little information on the potential vectors and maintenance hosts of rickettsiae is available in this region.

This study was conducted to determine the serologic status of Malaysian indigenous community and animal farm workers and provides molecular evidence regarding the presence of rickettsial agents in ticks/fleas infesting domestic animals in Peninsular Malaysia. Serum samples collected from urban blood donors were also included for comparison purpose. *R. conorii* and *R. felis* are the SFG rickettsiae that have been reported in most Asian countries including China,^12^ Korea,^13^ Laos,^14^ Taiwan^15^ and Thailand.^16^ Hence, the two rickettsial species were used as antigens in this serological assessment study. For vector surveillance, animal ectoparasites, mainly ticks and fleas, were collected from peri-domestic animals (cats, dogs, chickens, cattle and goats) from each study site for the detection of rickettsial DNA by using specific PCR assays followed by sequence analysis. It is hoped that the information derived from this study will be beneficial for surveillance, prevention and control of tick- and flea-borne rickettsioses in this region.

## MATERIALS AND METHODS

### Ethics statements

Ethical approval was obtained from the University Malaya Medical Centre (Ethics committee reference number: 944.20) for serological assessment of human serum samples. Prior to the commencement of the sample collection, permission was obtained from the Department of Orang Asli Development (JAKOA) and Department of Veterinary Services, Ministry of Agriculture and Agro-based Industry (DVS), Malaysia (reference number: JPV/PSTT/100-8/1). An oral briefing on the objective and methodology of the study was given to participants. Consent was obtained either in written form or verbally followed by thumb prints (for those who were illiterate). Parents or guardians of children under the age of 18 provided informed consent on their behalf. All data from the studied populations were strictly anonymized.

### Study population

Serum samples of 102 individuals residing at six rural villages who participated in a cross-sectional study (October 2012 to February 2013) to determine risk factors associated with dengue fever,^17^ and the seroprevalence of tick-borne viral diseases,^18^ were used. Details of the consent, sample collection, sampling scheme and population have been previously described.^17, 18^ The rural villages were mostly located at forest fringe areas and in close proximity to rubber or oil palm estates.

Serum samples were collected from 87 farm workers (February 2013 to September 2013) who were based on eight farms, designated as Farm 1–8 located in six states in Peninsular Malaysia; they included a cattle and a goat farm in Negeri Sembilan (*n*=24), two cattle farms in Pahang (*n*=18), one sheep farm in Kedah (*n*=7), and one cattle farm each in Kelantan (*n*=14), Terengganu (*n*=7) and Johore (*n*=17).^19^ For comparison, serum samples from 61 healthy blood donors residing in an urban area (Kuala Lumpur or Selangor) were kindly provided by the blood bank of the University Malaya Medical Centre for serological analysis.

### Serological analysis

The sera were analysed for lgG antibodies against *R. conorii* (strain Malish 7) and *R. felis* (strain LSU) using indirect immunofluorescence assay (IFA) kits (Fuller Laboratories, Fullerton, CA, USA) in accordance with the manufacturer's instructions. In brief, serum samples were first diluted (1:64) in phosphate-buffered saline (PBS) and 10 μL of each diluted serum sample was added to an antigen well on the IFA slide. After incubation at 37 °C for 30 min in a humidified chamber, the wells were washed with PBS and distilled water prior to incubation with IgG conjugate for 30 min. The slides were examined under × 400 magnification. Samples were regarded as positive when bright apple-green fluorescence of rickettsial antigens was observed (additional files; Supplementary Figures S1 and S2). The positive and negative sera provided in the IFA kits were used as controls. A past infection was indicated whenever there was an IgG titre of ≥1:64 without a fourfold or greater increase of titres.

### Statistical analysis

For comparison of seropositivity rates among different study groups, statistical analysis was performed using SPSS (Statistical Package for the Social Sciences) software program, version 22 (SPSS Inc., Chicago, IL, USA). Initial data entry was cross-checked (by KLK and MGK) in order to ensure that the data were entered correctly. *χ*^2^ and Kruskal–Wallis rank test were used to determine statistical significance between age, gender and study groups (indigenous population, animal farm workers and blood donors). The level of statistical significance was determined at *P*≤0.05 and 95% confidence interval (CI). Pairwise comparisons within the study and age groups were performed using Games–Howell *post hoc* tests of the SPSS software. A *P-*value of ≤0.05 was considered statistically significant.

### Collection of ticks and fleas

Ticks were collected using tweezers from the ear, eyes, flank, abdomen, tail and perineal regions of animals. Fleas were collected using combing method. Ticks were identified to the genus level according to Walker *et al.*^20^ and Geevarghese and Mishra.^21^ Molecular identification of tick species was performed using primers targeting tick 16S rRNA gene regions.^22^ All the fleas were identified as *Ctenocephalides felis* or *C. orientis* on the basis of morphometric characteristics.^23^ All the samples were preserved at −80 °C prior to DNA extraction.

### DNA extraction of tick/flea samples

The samples were processed according to Duh *et al.*^24^ with slight modification. In brief, ticks and fleas were first thawed and then immersed in 5% sodium hypochlorite and 70% ethanol before washing with sterile distilled water. The samples were then triturated using surgical blades and DNA was extracted using QIAamp DNA mini kits (Qiagen, Hilden, Germany) in accordance with the manufacturer's instruction.

### PCR amplification

Three rickettsial-specific genes, that is, citrate synthase gene (*gltA*),^25^ 190-kDa outer membrane protein gene (*ompA*)^26^ and 135-kDa outer membrane protein gene (*ompB*),^27^ were targeted for amplification from tick and flea samples. These PCR assays have been widely used for the detection of SFG rickettsiae in arthropod vectors.^28, 29, 30^ Due to the large sample size of cattle ticks and fleas, the *gltA* PCR assay was used for screening of rickettsial DNA. Any positive samples were then subjected to further amplification using primers targeting *ompA* and *ompB*. All three rickettsial PCR assays were used for detection of rickettsiae from ticks collected from rural villages and urban areas.

All PCR assays were performed in a final volume of 20 μL containing 2 μL of DNA template, × 1 ExPrime *Taq* DNA polymerase (GENET BIO, Daejeon, South Korea) and 0.2 μM of each primer, in a Veriti thermal cycler (Applied Biosystems, Foster City, CA, USA). DNA extracted from *R. conorii* antigen slides (Fuller Laboratories, Fullerton, CA, USA) was used as positive control for all the PCR assays. Sterile distilled water was used as the negative control in each PCR reaction. PCR products were purified using GeneAll Expin Combo GP kit (GeneAll Biotechnology, Seoul, Korea) prior to sequencing on an ABI PRISM 377 Genetic Analyzer (Applied Biosystems), using both forward and reverse primers of each PCR assay. The sequences obtained were subjected to BLAST analysis (http://blast.ncbi.nlm.nih.gov/Blast.cgi) to search for homologous sequences in the GenBank database.

### Phylogenetic analysis of tick- and flea-borne rickettsiae

To determine the phylogenetic placement of the rickettsiae identified in this study, a dendrogram was constructed based on *gltA* sequences (375 nucleotides) using the neighbour-joining method of MEGA software.^31^ Reference sequences for *R. raoultii*, *Rickettsia* sp. Rf31, *Rickettsia* sp. RF2125, *Candidatus* Rickettsia asemboensis, *Rickettsia* sp. California 2, *R. felis* URRWXCal2, *R. tamurae*, *Rickettsia* sp. *Kagoshima6*, *R. honei*, *R. conorii*, *R. heilongjiangensis*, *R. japonica* and *R. typhi* were retrieved from the GenBank database. Rickettsiae reported in previous Malaysian studies, including *Rickettsia* sp. clone HL2a, and clone HL15c, derived from cat fleas, *Rickettsia* sp. strain Mal from a febrile patient, and *Rickettsia* sp. 0095 from infected monkeys, were also included in the dendrogram.

## RESULTS

Table 1 presents the rickettsial seropositivity rates of different study groups based on gender and age. The median ages of 87 farm workers, 61 blood donors and 102 indigenous people were 38 years (range, 23 to 59 years), 31 years (range, 19 to 54 years) and 27 years (range, 8 to 78 years), respectively. The male-to-female ratio was 1.34 (143:107). The indigenous people had the highest seropositivity rates towards *R. conorii* (50.0%, 95% CI: 40.1%–59.9%) and *R. felis* (22.5%, 95% CI: 14.3%–30.8%). A total of 13.8% (95% CI: 6.4%–21.2%) and 16.1% (95% CI: 8.2%–24.0%) of the farm workers were seropositive for *R. conorii* and *R. felis*, respectively. The seropositivity rate for rickettsiae was the lowest among the urban blood donors, given that only 3.3% (95% CI: 0.0%–7.9%) were seropositive to *R. conorii* and none was seropositive for *R. felis*. Pairwise comparison within the study groups (using Games–Howell *post hoc* test) demonstrated a significantly higher *R. conorii*-seropositivity rate (50.0%±50.2%) in the indigenous people, compared with the animal farm workers (13.8%±34.7%, *P*<0.001) and the urban blood donors (3.3%±18.0%, *P*<0.001). The *R. felis*-seropositivity rate of the indigenous people (22.5%±42.0%) was also significantly higher compared with urban blood donors (0.0%, *P*<0.001), but not animal farm workers (16.1%±37.0%, *P*=0.500). Seropositivity against both *R. conorii* and *R. felis* was detected in 23 individuals (0 (0.0%) in urban blood donors, including 5 (5.7%) for farm workers and 18 (17.6%) for the indigenous community) in this study.

No significant differences in the seropositivity rates for *R. conorii* and *R. felis* were noted in any of the study groups based on gender (*P*=0.072 and *P*=0.509 for *R. conorii* and *R. felis*, respectively; Table 1). Significant differences were noted in the seropositivity rates for both *R. conorii* (*P*=0.011) and *R. felis* (*P*=0.001) within different age groups. The participants in the age group of ≥51 years old demonstrated the highest seropositivity rates to both *R. conorii* and *R. felis* (Table 1). Games–Howell *post hoc* tests revealed significantly higher *R. conorii*-seropositivity rate among participants over 50 years old (43.6%±50.2%) compared with those 41–50 years old (7.7%±27.0%, *P*=0.002). *R. felis*-seropositivity rate was also significantly higher among participants over 50 years old of age (33.3%±47.8%), compared with those ≤20 years old (5.4%±22.9%, *P*=0.015) and 31–40 years old (5.0%±22.0%, *P*=0.009).

The majority of the animal farm workers and urban blood donors were of the Malay ethnic group, which is the largest ethnic group in Malaysia, followed by Chinese and Indians. The indigenous people comprised different tribes including Temiar, Semoq Beri, Semai, Temuan, Jakun, Jah Hut, Kensui and others. Therefore, comparison of rickettsial-seropositivity rates between ethnic groups was not possible as each study group was composed of different ethnic group.

The tick/flea samples investigated in this study included:

270 ticks (70 *Rhipicephalus microplus* and 200 *Haemaphysalis bispinosa*) collected from cattle and sheep from eight animal farms. Each tick was processed individually.
186 ticks collected from 47 peri-domestic animals (which are cats, chickens, cattle, dogs and goats) from rural villages. The ticks (majority identified as *Haemaphysalis* spp.) were segregated into 64 pools (one to ten individuals) prior to DNA extraction. A total of 33 *Rh. sanguineus* collected from two animal shelters in urban area were also included.
153 fleas (42 *C. felis* and 111 *C. orientis*) infesting cats and dogs in rural villages and 210 fleas collected from stray cats in urban area (Kuala Lumpur).

Tables 2 and 3 summarize the results of PCR screening for rickettsial DNA from the ticks and fleas collected in this study. Rickettsial DNA was detected in 25 (9.3%) cattle ticks (21 *H. bispinosa* and 4 *Rh. microplus*) from four farms, with detection rates ranging from 2.1% to 27.7% (Table 2). BLAST analyzes were performed for 42 rickettsial sequences (20 *gltA*, 7 *ompA* and 15 *ompB*) obtained in this study (Supplementary Table S1). Sequence analyzes of the *gltA* fragments (375 bp) from 20 ticks reveal the identification of rickettsiae closely related to *R. raoultii* (*n*=15), *R. heilongjiangensis* (*n*=2), *Rickettsia* sp. RF2125 (*n*=1), *R. tamurae* (*n*=1) and *Rickettsia* sp. TCM1 (*n*=1). BLAST analysis of *ompA* (518 bp) in seven ticks (*H. bispinosa* from cattle) indicate the identification of a rickettsia closely related to *R. heilongjiangensis*. Nine and six of the *ompB* sequences (774–826 bp) matched those of *R. raoultii* and *Rickettsia* sp. RF2125, respectively (Supplementary Table S1).

The *gltA* sequences for the Malaysian *R. raoultii* strains demonstrated high sequence similarity (98%) to that of *R. raoultii* (GenBank accession NO: JQ 697956) reported from *H. hystricis* ticks in Japan. The *ompB* sequences demonstrated the highest identities (93%) to that of *R. raoultii* strain Khabarovsk (DQ365798), whereas the rickettsial *ompA* gene was not amplifiable. Further characterization is required to determine whether it represents a novel species of rickettsial species.

Of 186 ticks collected from peri-domestic animals in the rural villages (Table 2), rickettsial DNA was amplified from 40.6% (26/64) of the ticks (23 pools *Haemaphysalis* spp., one *Rh. sanguineus* and two *Rh. microplus*). Rickettsial-positive ticks were identified from nine rural villages, with the detection rates ranging from 14.3% to 66.7% in each village. A total of 43 sequences (13 *gltA*, 11 *ompA* and 19 *ompB*) were analyzed, and the BLAST results are presented in Supplementary Table S1. On the basis of sequence analysis, rickettsiae closely related to those of *R. tamurae* (98%), *R. felis* URRWXCal2 (99%), *Rickettsia* sp. RF2125 (98%–100%), *R. raoultii* (98%–99%) and *R. heilongjiangensis* (99%) were identified. In addition, a rickettsia identified from a *Haemaphysalis* cat tick from Kelantan shared 100% sequence similarity with the *gltA* sequence of *Rickettsia* sp. LON-13 (AB516964),^32^ whereas the *ompB* sequence derived from the tick resembled that of *R. hulinensis* (AY260452).^33^

Rickettsial DNA (either *gltA*, *ompA* and *ompB* gene fragments) was amplified from 39.4% (13/33) of *Rh. sanguineus* dog ticks in two animal shelters in Kuala Lumpur. The BLAST result reveals the identification of rickettsiae closely related to *Rickettsia* sp. RF2125, *R. conorii* type strains/*R. raoultii* strain Khabarovsk and *R. heilongjiangensis* (Supplementary Table S1).

A total of 60.1% (92/153) fleas collected from rural villages were positive for rickettsial DNA using both *gltA* and *ompB* PCR assays. Due to the large number of positive samples, only 22 amplified *gltA* and *ompB* fragments from different hosts and geographical locations were selected for sequence determination (Supplementary Table S2). The sequences were differentiated into two distinct types, of which one was more closely related to *R. feli*s strain URRWXCal2 (99%, 373/375 bp) for *gltA* and 100% (808/808 bp) for *ompB* sequences, GenBank accession no.: CP000053) and another one was more closely related to *Rickettsia* sp. RF2125 (99%, 373/374 bp) for *gltA* (GenBank accession no.: AF516333) and 100% (756/756 bp) for *ompB* sequences (GenBank accession no.: JX183538). Only 8.1% of *C. felis* collected from the urban area were positive in the rickettsial *gltA* PCR assays; however, the sequences were not determined due to insufficient amounts of amplified fragments.

The overall distribution of rickettsiae detected in the tick and flea samples and their animal hosts are summarized in Table 4. The detection rates of rickettsial DNA in animal ectoparasites varied from 0.0% to 66.7% for ticks and 0.0% to 96.9% for fleas in different locations (Tables 2 and 3). A dendrogram was constructed based on 19 *gltA* sequence types (375 bp) derived from ticks and fleas from different geographical regions in Peninsular Malaysia. The sequences were differentiated into two distinct groups: one closely related to the type strain of *R. felis* and another with the type strains of SFG rickettsiae.

**Figure 1:**
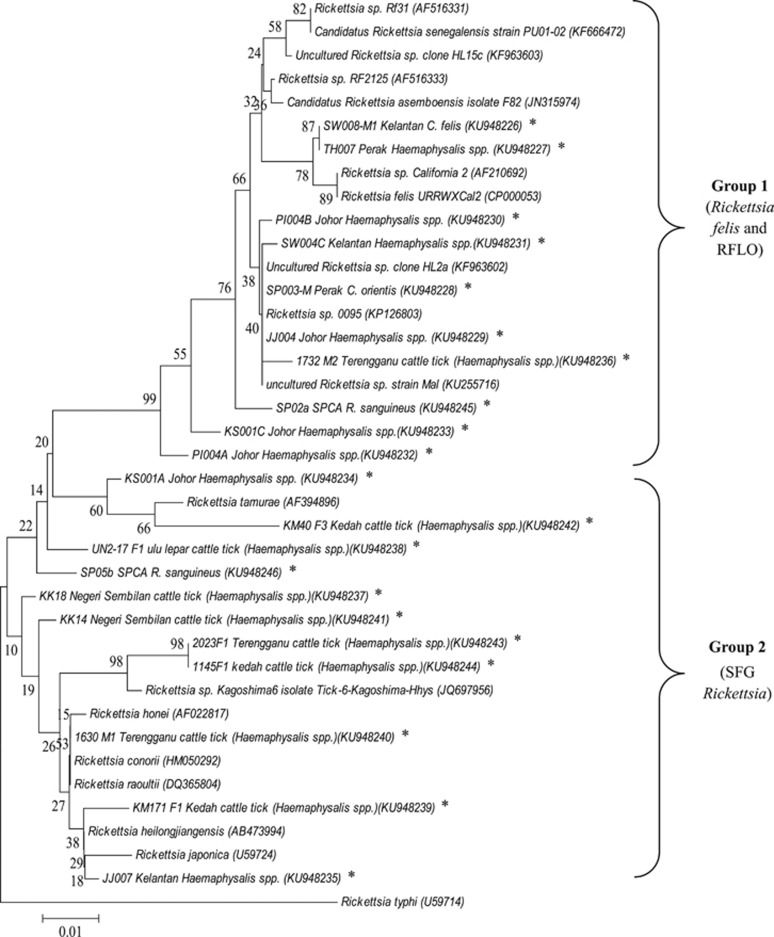
Phylogenetic placement of rickettsial *gltA* sequences (375 bp) amplified from ticks and fleas from different locations. The origins and details of the rickettsiae are presented in extended data (Supplementary Tables S1 and S2) and Table 4. Bootstrap analysis was performed with 1000 replications. The scale bar indicates the nucleotide substitutions per site. * indicates rickettsiae detected in this study.

In this study, eight *gltA* sequence variants were identified in the *R. felis* group (exhibiting 2–13 nucleotide differences), as compared to that of *R. felis* strain URRWXCal2 (CP000053; Figure 1). One matched 99% to that of *R. felis* strain URRWXCal2 and the remaining seven sequence variants matched 97%–99% with the *Rickettsia* sp. RF2125 (AF516333). One matched 100% with the uncultured *Rickettsia* sp. clone-4-G/JP-10-2 reported in dog flea in Guatemala and Costa Rica (JN982949).^34^ Owing to the close sequence similarity between the SFG rickettsiae, low bootstrap values were noted between branches in the dendrogram (Figure 1). The *gltA* sequences derived from this study have been deposited in the GenBank database: KU948226 – KU948246.

## DISCUSSION

This study provides an updates on the exposure of Malaysian indigenous community and animal farm workers to rickettsioses through serological assessment against *R. conorii* and *R. felis*. Molecular detection of rickettsiae was also conducted in ticks and fleas infesting domestic animals in the respective surveyed areas to identify possible rickettsial agents that were circulating in our environment. Antigenic cross-reactivity has been reported among SFG rickettsiae.^2, 35^ The cross-reactivity between members of SFG rickettsiae, including *R. conorii*, *R. rickettsii*, *R. helvetica*, *R. slovaca*, *R. massiliae*, *R. africae* and others has been highlighted by the manufacturer. Hence, it is possible that IgG for other SFG rickettsiae members to be present in the participants of this study. Similarly, cross-reactivity between *R. felis* and TG *Rickettsia*, and *R. akari* and *R. australis* was also stated by the manufacturer in the brochure. Znazen *et al.*^36^ hypothesized that many reactions due to *R. conorii* could be caused by *R. felis*. In this study, individuals seropositive for both rickettsial species were noted of the 5.7% of farm workers, 17.6% of the indigenous community and none of the urban blood donors. However, it is difficult to differentiate *R. felis* and other SFG rickettsia without the use of further serological assays, such as cross-absorption techniques and western blot.^35^

Our findings indicate that SFG rickettsioses are prevalent in the indigenous community. Up to 50% of the individuals exhibit seropositivity against *R. conorii*, whereas approximately one-fourth of the population was previously exposed to *R. felis*. The seroprevalence of rickettsioses is affected by the geographical differences, lifestyle and occupation of subjects investigated.^9^ According to a recent survey by Chandren *et al.*,^17^ a majority of the indigenous people in Malaysia lived in wooden houses or simple cement homes in close proximity to jungle and plantation areas, which expose them to infected animal ectoparasites such as ticks and fleas. Their close contact with animals and work environment enhances the risk of contracting tick- and flea-borne diseases. In addition, there is a lack of awareness about rickettsioses that hampers the prevention practices in the community. The seropositivity to *R. conorii* in urban blood donors was relatively low (3.3%) compared with that obtained from a previous serosurvey (1.7%).^4^

Farm workers may be subjected to increasing risk of tick- and flea-borne diseases.^37^ For instance, exposures to several tick- and flea-borne pathogens have been reported among farm workers in Tianjin, China.^11^ The presence of various SFG rickettsiae and *R. felis* in cattle ticks (*H. bispinosa* and *Rh. microplus*) was demonstrated in the vector surveillance in this study (Table 4). Although the vectorial capacity of the infected ticks is yet to be established, the results in this study highlight the potential exposure of farm workers to rickettsioses. Higher seropositivity rates against *R. conorii* and *R. felis* were observed in older age group (>50 years old), compared with younger age groups (Table 1) in this study. This result could be due to long-term persistence of antibody, as also noted for scrub typhus in Malaysia.^9^ A low seroprevalence (3.3% and 0.0% for *R. conorii* and *R. felis*, respectively) was noticed in urban blood donors, and this finding could be due to low exposure of the urban population or different species of ticks found compared with those in the rural areas. In addition, the occurrence of *Rickettsia* spp. in fleas collected from urban areas is significantly lower compared with the fleas collected from rural areas (Table 3) and this result may be an explanation for the relatively lower seropositivity observed in urban blood donors.

Recent investigations have demonstrated the prevalence of SFG rickettsioses in Southeast Asia. A relatively high seropositivity rate of SFG rickettsial infection has been reported in Thai patients from Chiangrai (33.0%) and Mae Sot (27.3%), who presented with undifferentiated febrile illness.^38^ A seroprevalence of 20.4% towards *R. conorii* has also been reported in healthy rural residents from Gag Island, Indonesia.^39^ Sequence analysis of amplified fragments of *gltA*, *ompA* and *ompB* genes from ticks and fleas collected in this study shows the identification of a number of rickettsiae that had been previously reported, including *R. raoultii*, *R. tamurae*, *Rickettsia* sp. TCM1 and *Rickettsia* sp. RF2125 (Table 4). In addition to these rickettsial species, a *R. heilongjiangensis*-like organism was detected for the first time from *Haemaphysalis* ticks in cattle, cats, chickens, dogs and *Rh. sanguineus* from dogs in urban area. This rickettsia is distributed in the Russian Far East and Northern China. Recently, a phylogenetically related strain (PMK94) was isolated from a patient with septic shock in Thailand.^40, 41^

Since the first report of *R. felis* infection among rural residents of the central Thai Myanmar border,^42^ *R. felis* has been identified in febrile patients in several Asian countries, including Korea, Thailand, and Laos.^35^ A *R. felis*-like organism (RFLO) was detected in a febrile patient,^43^ cat fleas and cynomolgus monkeys in recent Malaysian studies.^44, 45, 46^ The findings in this study indicate that 22.5% of the indigenous populations and 16.1% of farm workers were previously been exposed to *R. felis*. In contrast, none of the urban blood donors tested was seropositive to *R. felis* (Table 1). The *R. felis-*seropositivity rate (16.1%) in our farm workers was similar to that reported for healthy individuals in Jiangsu province, China.^11^ In a recent study in Spain, higher seroprevalence of *R. felis* in rural areas (7.1%) compared with urban (3.5%) and semirural area (1.7%) was reported.^47^ In this study, the higher *R. felis* seropositivity in the indigenous community correlates with higher detection rates of *R. felis*/RFLO in fleas collected from rural areas as compared with urban areas (Table 3). The detection of *R. felis/*RFLO from fleas (*C. felis* and *C. orientis*), and various tick species (*Haemaphysalis* spp., *Rh. microplus* and *Rh. sanguineus*) collected from cattle, sheep, chickens, cats and dogs from different study sites in Peninsular Malaysia (Table 4), suggests the widespread existence of the rickettsial organism.

Several studies reported the presence of rickettsiae in cattle ticks in the Asia-Pacific region. Uncharacterized *Rickettsia* sp. has been reported in *H. longicornis* (12.4%) from grazing cattle in Korea.^48^ Rickettsiae exhibited high sequence similarities with *R. heilongjiangensis*, and *Rickettsia* sp. LON-13 was identified in *Rh. microplus* in Laos.^49^ In northeastern China, rickettsiae exhibiting a close phylogenetic relationship with *R. raoultii* (0.6%) and *R. japonica* (3.3%) was reported in *H. longicornis* ticks collected from domestic animals (sheep and cattle).^50^ In this study, rickettsiae closely related to *R. raoultii*, *R. heilongjiangensis* and *R. felis*/RFLO were identified from some cattle ticks (Table 4). All these findings suggest that cattle ticks could be a potential maintenance host for rickettsiae; however, further investigation is required to determine the vectorial capability of the ticks.

In a recent Malaysian study, a rickettsia closely related to *R. raoultii* has been implicated as the aetiological agent for rickettsioses in two febrile patients.^43^ *R. raoultii*, the causative agent for tick-borne lymphadenopathy, was reported in *Haemaphysalis* ticks from Thailand and is widely distributed in *Dermacentor* ticks in northern China.^51, 52, 53^ In addition to cattle ticks, this study also reports the identification of closely related strains of *R. raoultii* in *Rh. sanguineus* and *Haemaphysalis* ticks infesting peri-domestic animals in the rural villages (Table 4). Previously, closely related strains of *R. raoultii* were reported in Malaysian wild rats^45^ and *Amblyomma* spp. parasitizing wild snakes.^54^

*Rickettsia* sp. LON-13 (closely related to *R. japonica*)^55^ was reported for the first time from a *Haemaphysalis* cat tick in this study. A mixed rickettsial infection was suspected in the *Haemaphysalis* cat tick as *R. hulinensis* (first isolated from *H. concinna* ticks collected in Hulin Country, China),^56^ was also detected from the same tick through sequence analysis of the *ompB* sequence. In fact, based on BLAST analyses of rickettsial *gltA*, *ompA* and *ompB* gene sequences in this study, the presence of more than one rickettsial organism in a single tick was noted in this study.

*Rh. sanguineus*, a three-host tick mainly infesting dogs, is the main reservoir of *R. conorii*.^57^ The vector surveillance in this study indicates that *Rh. sanguineus* was the only tick species recovered from urban dogs in this study. The detection of a rickettsia closely related to RFLO (resembling *Rickettsia* sp. RF2125) in *Rh. sanguineus* dog ticks, has also been reported in a Chinese study.^58^

Taken together, this study provides a glimpse of the serological status of Malaysian indigenous people and farm workers against rickettsioses. Some potential limitations of the vector surveillance study are addressed here. For instance, as rickettsiae were detected mainly by PCR approach, it is possible that some might have remained undetected due to the bias of PCR assays in amplifying certain rickettsiae.^59^ Hence, other microbial detection methods should be used to complement the findings, especially when more than one type of rickettsial species is present in the tick or flea samples. The species status of rickettsiae should be confirmed by isolation of the rickettsiae. Extensive studies should be conducted on a larger sample sizes in multi-locations to assess the correlation between the rickettsia in ticks/fleas and human seropositivity in urban area. Determination of the vectorial capacity of ticks and fleas is necessary to illustrate the involvement of these ectoparasites in the transmission of rickettsioses.

On the basis of serological data obtained in this study, infections due to *R. conorii* and *R. felis* appear to be a health concern to the Malaysian indigenous community and farm workers. The data obtained from vector surveillance in this study would be helpful for the public health authority in formulating prevention and control strategies for rickettsioses.

## Figures and Tables

**Table 1:** Seropositivity of *R. conorii* and *R. felis* with respect to different category of the participants investigated in this study

**Categories**	***R. conorii***			***R. felis***		
	**Number (%)**	***P-*value**	**95% CI**	**Number (%)**	***P-*value**	**95% CI**
*Study group*						
Blood donors (*n*=61)	2 (3.3)^a^	<0.001	0.0%–7.9%	0 (0.0)^a^	<0.001	0.0%–0.0%
Farm workers (*n*=87)	12 (13.8)^a^		6.4%–21.2%	14 (16.1)		8.2%–24.0%
Indigenous people (*n*=102)	51 (50.0)		40.1%–59.9%	23 (22.5)		14.3%–30.8%

*Gender*						
Male (*n*=143)	31 (21.7)	0.072	14.8%–28.5%	23 (16.1)	0.509	10.0%–22.2%
Female (*n*=107)	34 (31.8)		22.8%–40.7%	14 (13.1)		6.6%–19.6%

*Age group (years)*						
≤20 (*n*=37)	10 (27.0)	0.011	12.0%–42.0%	2 (5.4)^b^	0.001	0.0%–13.0%
21–30 (*n*=75)	19 (25.3)		15.3%–35.4%	11 (14.7)		6.5%–22.9%
31–40 (*n*=60)	16 (26.7)		15.1%–38.2%	3 (5.0)^b^		0.0%–10.7%
41–50 (*n*=39)	3 (7.7)^b^		0.0%–16.4%	8 (20.5)		7.3%–33.8%
≥51 (*n*=39)	17 (43.6)		27.3%–59.9%	13 (33.3)		17.9%–48.8%

Abbreviation: confidence interval, CI.

a Significant difference in the rickettsial-seropositivity rate when compared to the indigenous people (Games–Howell *post hoc* test).

b Significant difference in the rickettsial-seropositivity rate when compared to those ≥51 years of age (Games–Howell *post hoc* test).

**Table 2:** Seroprevalences of *R. conorii* and *R. felis* among animal farm workers, indigenous people and blood donors in different localities; information on the tick species and detection rates of rickettsiae in each locality are also included

**Localities**	**Number (%, 95% CI) of people seropositive to *R. conorii***	**Number (%, 95% CI) of people seropositive to *R. felis***	**Animal host examined (*n*)**	**Ticks species (*n*)**	**Number (%, 95% CI) of ticks with rickettsia detection**
*Farms*					
Negeri Sembilan (Farm 1)	3 (17.7, 0.0%–37.9%)	6 (35.3, 10.0%–60.6%)	Cattle (30)	*H. bispinosa* (41) and *Rh. microplus* (6)	13 (27.7, 14.4%–40.9%)
Pahang (Farm 2)	0 (0.0, 0.0%–0.0%)	0 (0.0, 0.0%–0.0%)	Cattle (39)	*Rh. microplus* (14)	0 (0.0, 0.0%–0.0%)
Pahang (Farm 3)	5 (45.5, 10.4%–80.5%)	2 (18.2, 0.0%–45.4%)	Cattle (40)	*H. bispinosa* (57) and *Rh. microplus* (37)	2 (2.1, 0.0%–5.1%)
Kedah (Farm 4)	0 (0.0, 0.0%)	2 (28.6, 0.0%–73.7%)	Sheep (40)	*H. bispinosa* (44)	7 (15.9, 4.7%–27.2%)
Kelantan (Farm 5)	2 (14.3, 0.0%–35.3%)	1 (7.1, 0.0%–22.6%)	Cattle (40)	Not determined	—
Terengganu (Farm 6)	0 (0.0, 0.0%–0.0%)	2 (28.6, 0.0%–73.7%)	Cattle (40)	*H. bispinosa* (58) and *Rh. microplus* (13)	3 (4.2, 0.0%–9.0%)
Negeri Sembilan (Farm 7)	0 (0.0, 0.0%–0.0%)	0 (0.0, 0.0%–0.0%)	Goat (40)	0	0 (0.0, 0.0%–0.0%)
Johore (Farm 8)	2 (11.8, 0.0%–28.8%)	1 (5.9, 0.0%–18.4%)	Dairy cattle (41)	0	0 (0.0, 0.0%–0.0%)
Total	12 (13.8, 6.4%–21.2%)	14 (16.1, 8.2%–24.0%)		270 individual ticks	25 (9.3, 5.8%–12.7%)

*Rural area*					
Negeri Sembilan	—	—	Cat (12), chicken (1), dog (40), goat (8)	*Rh. sanguineus*, *Haemaphysalis* spp. (3 individuals and 4 pools)	4 (57.1, 7.7%–100.0%)
Pahang	5 (15.2, 2.2%–28.1%)	5 (15.2, 2.2%–28.1%)	Cat (18), chicken (5), dog (21),	*Haemaphysalis* spp. (7 pools)	1 (14.3, 0.0%–49.2%)
Kedah	—	—	Cat (17), chicken (1), cattle (3), dog (16)	*Rh. microplus* (3 individuals)	2 (66.7, 0.0%–100.0%)
Kelantan	39 (79.6, 67.9%–91.3%)	13 (26.5, 13.7%–39.3%)	Cat (27), chicken (9), dog (4)	*Haemaphysalis* spp. (10 individuals and one pool)	7 (63.6, 29.7%–97.5%)
Johore	7 (35.0, 12.1%–57.9%)	5 (25.0, 4.2%–45.8%)	Cat (23), chicken (2), dog (15)	*Haemaphysalis* spp. (one individual and 8 pools)	5 (55.6, 15.0%–96.1%)
Perak	—	—	Cat (8), chicken (2), dog (10)	*Haemaphysalis* spp. (16 individuals and 11 pools)	7 (25.9, 8.3%–43.6%)
Total	51 (50.0, 40.1%–59.9%)	23 (22.5, 14.3%–30.8%)		33 individuals and 31 pools	26 (40.6, 28.3%–53.0%)

*Urban area*					
Animal shelters	—	—	Dog (13)	*Rh. sanguineus* (33 individuals)	13 (39.4, 21.8%–57.0%)
Blood donors	2 (3.3, 0.0%–7.9%)	0 (0.0, 0.0%)	—	—	—

Abbreviation: confidence interval, CI.

**Table 3:** The detection rates of rickettsiae from fleas collected in each locality

**Localities**	**Animal host (*n*)**	**Flea species**	**Number of fleas tested**	**Number (%, 95% CI) of fleas with rickettsia detection**
*Rural area*				
Negeri Sembilan	Dog (19), cat (3)	*C. orientis*, *C. felis*	36	26 (72.2, 56.9%–87.6%)
Pahang	Dog (16)	*C. orientis*	26	15 (57.7, 37.3%–78.0%)
Kedah	Cat (7)	*C. felis*	14	0 (0.0, 0.0%–0.0%)
Kelantan	Cat (13)	*C. felis*, *C. orientis*	26	7 (26.9, 8.7%–45.2%)
Johore	Dog (9)	*C. orientis*	32	31 (96.9, 90.5%–100.0%)
Perak	Dog (9), cat (1)	*C. orientis*, *C. felis*	19	13 (68.4, 45.4%–91.4%)
Total			153	92 (60.1, 52.3%–68.0%)

*Urban area*				
DBKL	Cat (18)	*C. felis*	162	17 (10.5, 5.7%–15.3%)
Titiwangsa	Cat (18)	*C. felis*	48	0 (0.0, 0.0%–0.0%)
Total			210	17 (8.1, 4.4%–11.9%)

Abbreviations: confidence interval, CI; Dewan Bandaraya Kuala Lumpur, DBKL.

**Table 4:** Overall distribution of rickettsiae detected in ticks/fleas and their animal hosts in each location

**Rickettsial species**	**Tick/flea species**	**Animal host**	**Location**
*R. raoultii*-like	*H. bispinosa*	Cattle	Negeri Sembilan, Terengganu
	*H. bispinosa*	Sheep	Kedah
	*Haemaphysalis* spp.	Chicken	Negeri Sembilan, Pahang, Perak
	*Haemaphysalis* spp.	Dog	Negeri Sembilan, Perak
	*Haemaphysalis* spp.	Cat	Perak
	*Rh. microplus*	Cattle	Kedah, Pahang
	*Rh. sanguineus*	Dog	Negeri Sembilan, Kuala Lumpur
*R. heilongjiangensis*-like	*H. bispinosa*	Cattle	Negeri Sembilan
	*Haemaphysalis* spp.	Cat	Kelantan, Johore
	*Haemaphysalis* spp.	Chicken	Kelantan, Johore
	*Haemaphysalis* spp.	Dog	Johore
	*Rh. microplus*	Cattle	Negeri Sembilan
	*Rh. microplus*	Cattle	Pahang
	*Rh. sanguineus*	Dog	Kuala Lumpur
*Rickettsia*-like organisms (RFLO)	*C. orientis*	Cat	Johore, Pahang, Perak, Negeri Sembilan
	*H. bispinosa*	Cattle	Negeri Sembilan, Terengganu
	*H. bispinosa*	Sheep	Kedah
	*Haemaphysalis* spp.	Cat	Kelantan, Johore
	*Haemaphysalis* spp.	Chicken	Kelantan, Johore
	*Haemaphysalis* spp.	Dog	Johore
	*Rh. microplus*	Cattle	Negeri Sembilan
	*Rh. sanguineus*	Dog	Kuala Lumpur
*R. tamurae*-like	*H. bispinosa*	Sheep	Kedah
	*Haemaphysalis* spp.	Cat	Johore
*Rickettsia* sp. TCM1	*H. bispinosa*	Sheep	Kedah
*R. felis* URRWXCal2	*C. felis*	Cat	Kelantan
	*Haemaphysalis* spp.	Dog	Perak
	*Rh. microplus*	Cattle	Kedah
*Rickettsia* sp. LON-13	*Haemaphysalis* spp.	Cat	Kelantan
*R. hulinensis*	*Haemaphysalis* spp.	Cat	Kelantan
