# Supplementary material for: Rickettsial seropositivity in the indigenous community and animal farm workers, and vector surveillance in Peninsular Malaysia
Source: Emerg Microbes Infect. 2017 Apr 12;6(4):e18–. doi: 10.1038/emi.2017.4 (PMC5457682; doi:10.1038/emi.2017.4)
Supplement: Supplementary Figure 2 [file emi20174x2.pdf]

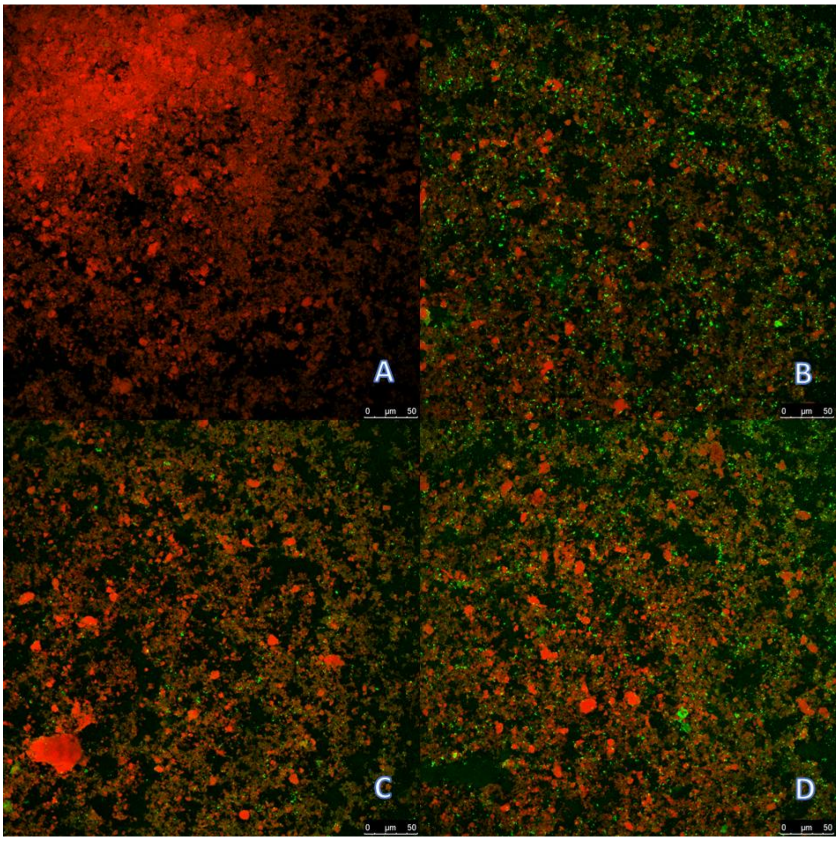

Supplementary Figure S2 Representative images of *R. felis* immunofluorescence assay (Fuller Laboratories, USA). **(A)** Negative control, **(B)** Positive control, **(C)** positive sample from farm worker, and **(D)** positive sample from rural villager. Analysis was performed in a Leica TCS SP5 II laser scanning confocal spectral microscope with a 40x oil immersion objective len. Scale bar: 50 µm.
