# Supplementary material for: Rickettsial seropositivity in the indigenous community and animal farm workers, and vector surveillance in Peninsular Malaysia
Source: Emerg Microbes Infect. 2017 Apr 12;6(4):e18–. doi: 10.1038/emi.2017.4 (PMC5457682; doi:10.1038/emi.2017.4)
Supplement: Supplementary Table 1 [file emi20174x3.docx]

Supplementary Table S1 Blast analysis of rickettsial *gltA*, *ompA* and *ompB* gene fragments of ticks from animal farms, rural villages and urban areas.

| Localities | Tick species positive for *Rickettsia* (n; host) | BLAST analysis (closest relative) | | |
| --- | --- | --- | --- | --- |
|  |  | *gltA* [n] | *ompA* [n] | *ompB* [n] |
| **Animal farms** | | | | |
| Negeri Sembilan (Farm 1) | *H. bispinosa* (11; cattle) | *Rickettsia* *raoultii* (JQ697956; 366/375 (98%)); [6] | N/A | *Rickettsia raoultii* strain Khabarovsk (DQ365798; 740/800(93%)); [5] |
|  |  | *Rickettsia heilongjiangensis* type strains (393-373/375(99%)); [1] | N/A | N/A |
|  |  | *Rickettsia heilongjiangensis* type strains (373/375(99%)); [1] | *Rickettsia heilongjiangensis* (CP002912; 500/506(99%)); [1] | *Rickettsia* sp. RF2125 (JX183538, 790/790 (100%)); [1] |
|  |  | N/A | *Rickettsia heilongjiangensis* (CP002912; 500/506(99%)); [3] | *Rickettsia* sp. RF2125 (JX183538, 790/790 (100%)); [2] |
|  | *Rh. microplus* (2; cattle) | N/A | *Rickettsia heilongjiangensis* (CP002912; 500/506(99%); [2] | *Rickettsia* sp. RF2125 (JX183538, 790/790 (100%)); [2] |
| Pahang (Farm 3) | *Rh. microplus* (2; cattle) | *Rickettsia conorii* type strains /*Rickettsia raoultii* type strains, (370-375/375(99-100%)); [2] | *Rickettsia heilongjiangensis* (CP002912; 500/506(99%); [1] | N/A |
| Kedah (Farm 4) | *H. bispinosa* (7; sheep) | *Rickettsia* *raoultii* ( JQ697956; 366/375 (98%)); [5] | N/A | *Rickettsia raoultii* strain Khabarovsk (DQ365798; 740/800(93%)); [3] |
|  |  |  |  | *Rickettsia* sp. RF2125 (JX183538, 790/790 (100%)); [1] |
|  |  | *Rickettsia tamurae* strain AT-1 (AF394896), (365/375 (97%)); [1] | N/A | N/A |
|  |  | *Rickettsia* sp. TCM1 (B359458; 374/375(99%)), [1] | N/A | N/A |
| Terengganu (Farm 6) | *H. bispinosa* (3; cattle) | *Rickettsia* *raoultii* (JQ697956; 366/375 (98%)); [1] | N/A | *Rickettsia raoultii* strain Khabarovsk (DQ365798; 740/800(93%)); [1] |
|  |  | *Rickettsia* sp. RF2125 (AF516333, 392/397(99%)); [1] | N/A | N/A |
|  |  | *Rickettsia conorii* type strains /*Rickettsia raoultii* type strains, (375/375(100%)); [1] | N/A | N/A |
| **Rural area** | | | | |
| Negeri Sembilan | *Rh. sanguineus* (1; dog) | N/A | N/A | *Rickettsia raoultii* strain Khabarovsk (DQ365798, 790/802(99%)); [1] |
|  | *Haemaphysalis* spp. (2; 1 chicken and dog, respectively) | N/A | N/A | *Rickettsia raoultii* strain Khabarovsk (DQ365798, 787-789/802(98%)); [2] |
| Pahang | *Haemaphysalis* spp. (1; chicken) | N/A | N/A | *Rickettsia raoultii*  strain Khabarovsk (DQ365798, 787-787/802(98%)); [1] |
| Kedah | *Rh. microplus* (2; cattle) | *Rickettsia felis* URRWXCal2 (CP000053, 369/370(99%)); [1] | N/A | *Rickettsia felis* URRWXCal2 (CP000053, 807/808 (99%)); [1] |
|  |  | N/A | N/A | *Rickettsia raoultii* strain Khabarovsk (DQ365798, 790/802(99%)); [1] |
| Kelantan | *Haemaphysalis* spp. (8; 4 chicken and cats, respectively) | *Rickettsia* sp. LON-13 (AB516964, 375/375(100%)); [1] | N/A | *Rickettsia hulinensis* (AY260452, 776/802(97%)); [1] |
|  |  | *Rickettsia* sp. RF2125 (AF516333, 373-374/376(99%)); [6] | *Rickettsia heilongjiangensis* (CP002912; 500/506(99%); [5] | N/A |
|  |  | N/A | *Rickettsia heilongjiangensis* (CP002912; 500/506(99%); [1] | N/A |
| Johore | *Haemaphysalis* spp. (5; 2 dogs, a cat and chicken, respectively) | *Rickettsia tamurae* (AB812551, 366/375(98%)); [1] | *Rickettsia heilongjiangensis* (CP002912; 500/506(99%); [1] | *Rickettsia* sp. RF2125 (JX183538, 756/756 (100%)); [1] |
|  |  | *Rickettsia* sp. RF2125 (AF516333, 367/376(98%)); [1] | *Rickettsia heilongjiangensis* (CP002912; 500/506(99%); [1] | *Rickettsia* sp. RF2125 (JX183538, 756/756 (100%)); [1] |
|  |  | *Rickettsia* sp. RF2125 (AF516333, 373/376(99%); [2] | *Rickettsia heilongjiangensis* (CP002912; 500/506(99%); [2] | *Rickettsia* sp. RF2125 (JX183538, 756/756 (100%)); [2] |
|  |  | N/A | *Rickettsia heilongjiangensis* (CP002912; 500/506(99%); [1] | *Rickettsia* sp. RF2125 (JX183538, 756/756 (100%)); [1] |
| Perak | *Haemaphysalis* spp. (7; 2 dogs and 5 cats) | *Rickettsia felis* URRWXCal2 (CP000053, 369/370(99%)); [1] | N/A | *Rickettsia felis* URRWXCal2 (CP000053, 807/808 (99%); [1] |
|  |  | N/A | N/A | *Rickettsia raoultii* strain Khabarovsk (DQ365798, 789-790/802(98-99%)); [6] |
| **Urban area** | | | | |
| Animal shelter, Kuala Lumpur | *Rh. sanguineus* (13 positive, 6 representative sequenced; dogs) | N/A | *Rickettsia heilongjiangensis* (CP002912; 511/518 (99%)); [2] | *Rickettsia raoultii* strain Khabarovsk (DQ365798, 790/802(99%)), [2] |
|  |  | *Rickettsia* sp. RF2125 (AF516333, 369/375 (98%)); [1] | N/A | *Rickettsia raoultii* strain Khabarovsk (DQ365798, 790/802(99%)), [ 1] |
|  |  | N/A | *Rickettsia heilongjiangensis* (CP002912; 511/518 (99%)); [1] | *Rickettsia* sp. RF2125 (JX183538, 752/756 (99%)); [1] |
|  |  | *Rickettsia conorii* type strains/ *Rickettsia raoultii* strain Khabarovsk (DQ365804, 369/375(98%)); [1] | *Rickettsia heilongjiangensis* (CP002912; 511/518 (99%)); [1] | *Rickettsia* sp. RF2125 (JX183538, 756/756 (100%)); [1] |
|  |  | *Rickettsia conorii* type strains/ *Rickettsia raoultii* strain Khabarovsk (DQ365804, 369/375(98%)); [1] | N/A | N/A |
