# Supplementary material for: Rickettsial seropositivity in the indigenous community and animal farm workers, and vector surveillance in Peninsular Malaysia
Source: Emerg Microbes Infect. 2017 Apr 12;6(4):e18–. doi: 10.1038/emi.2017.4 (PMC5457682; doi:10.1038/emi.2017.4)
Supplement: Supplementary Table 2 [file emi20174x4.docx]

Supplementary Table S2 Blast analysis of the selected rickettsial *gltA* and *ompB* gene sequences from fleas from rural villages.

| **Location (Number of *Rickettsia* positive fleas)** | **Number of samples sequenced** | BLAST analysis (closest relative) | |
| --- | --- | --- | --- |
|  |  | ***gltA*** | ***ompB*** |
| ***C. felis*** | | | |
| SW, Kelantan (5) | 2 | *R. felis* URRWXCal2 (CP000053, 373/375(99%)) | *R. felis* URRWXCal2  (CP000053, 808/808(100%)) |
| JJ, Kelantan (1) | 1 |  |  |
| ***C. orientis*** | | | |
| JJ, Kelantan (1) | 1 | *Rickettsia* sp. RF2125 (AF516333, 373/375 (99%)) | *Rickettsia* sp. RF2125 (JX183538, 756/756 (100%)) |
| SD, Johore (31) | 5 |  |  |
| PP, Pahang (5) | 2 |  |  |
| PS, Pahang (10) | 2 |  |  |
| TH, Perak (7) | 2 |  |  |
| SP, Perak (6) | 2 |  |  |
| Negeri Sembilan (26) | 5 |  |  |
